# SmartFFR, a New Functional Index of Coronary Stenosis: Comparison With Invasive FFR Data

**DOI:** 10.3389/fcvm.2021.714471

**Published:** 2021-08-17

**Authors:** Panagiotis K. Siogkas, Lampros Lakkas, Antonis I. Sakellarios, George Rigas, Savvas Kyriakidis, Kostas A. Stefanou, Constantinos D. Anagnostopoulos, Alberto Clemente, Silvia Rocchiccioli, Gualtiero Pelosi, Oberdan Parodi, Michail I. Papafaklis, Katerina K. Naka, Lampros K. Michalis, Danilo Neglia, Dimitrios I. Fotiadis

**Affiliations:** ^1^Biomedical Research Institute, Foundation for Research and Technology Hellas, Ioannina, Greece; ^2^Materials Science and Engineering, University of Ioannina, Ioannina, Greece; ^3^Faculty of Medicine, School of Health Sciences, University of Ioannina, Ioannina, Greece; ^4^PET-CT Department & Preclinical Imaging Unit, Center for Experimental Surgery, Clinical & Translational Research, Biomedical Research Foundation Academy of Athens, Athens, Greece; ^5^Fondazione Toscana G. Monasterio and Institute of Clinical Physiology, Consiglio Nazionale delle Ricerche, Pisa, Italy; ^6^Institute of Clinical Physiology, CNR, Pisa, Italy

**Keywords:** cardiovascular diseases, coronary stenosis, functional assessment, smartFFR, atherosclerosis

## Abstract

**Aims:** In this study, we evaluate the efficacy of SmartFFR, a new functional index of coronary stenosis severity compared with gold standard invasive measurement of fractional flow reserve (FFR). We also assess the influence of the type of simulation employed on smartFFR (i.e. Fluid Structure Interaction vs. rigid wall assumption).

**Methods and Results:** In a dataset of 167 patients undergoing either computed tomography coronary angiography (CTCA) and invasive coronary angiography or only invasive coronary angiography (ICA), as well as invasive FFR measurement, SmartFFR was computed after the 3D reconstruction of the vessels of interest and the subsequent blood flow simulations. 202 vessels were analyzed with a mean total computational time of seven minutes. SmartFFR was used to process all models reconstructed by either method. The mean FFR value of the examined dataset was 0.846 ± 0.089 with 95% CI for the mean of 0.833–0.858, whereas the mean SmartFFR value was 0.853 ± 0.095 with 95% CI for the mean of 0.84–0.866. SmartFFR was significantly correlated with invasive FFR values (R_CCTA_ = 0.86, *p*_CCTA_ < 0.0001, R_ICA_ = 0.84, *p*_ICA_ < 0.0001, *R*_overall_ = 0.833, *p*_overall_ < 0.0001), showing good agreement as depicted by the Bland-Altman method of analysis. The optimal SmartFFR threshold to diagnose ischemia was ≤0.83 for the overall dataset, ≤0.83 for the CTCA-derived dataset and ≤0.81 for the ICA-derived dataset, as defined by a ROC analysis (AUC_overall_ = 0.956, *p* < 0.001, AUC_ICA_ = 0.975, *p* < 0.001, AUC_CCTA_ = 0.952, *p* < 0.001).

**Conclusion:** SmartFFR is a fast and accurate on-site index of hemodynamic significance of coronary stenosis both at single coronary segment and at two or more branches level simultaneously, which can be applied to all CTCA or ICA sequences of acceptable quality.

## Introduction

Fractional flow reserve (FFR) is considered the gold standard for the assessment of the severity of coronary stenoses in patients undergoing invasive coronary angiography (ICA). It can reliably recognize hemodynamically significant lesions, thus providing an excellent tool for percutaneous coronary intervention (PCI) guidance (1, 2). However, FFR is not widely used in the clinical settings probably due to its increased total procedural cost (>﹩1,000 average cost per patient, including the dedicated pressure wire and the vasodilator adenosine administered i.v. to induce hyperemia), as well as the added discomfort of the patient. In fact, in the evaluation of intermediate stenoses (i.e. 40–70%) prior to intervention, FFR is measured only in 6.1% of the cases (3).

Accordingly, alternative invasive and non-invasive coronary functional assessment methods based on computational models have been proposed not requiring pressure measurements and vasodilator administration. During the past decade, computed tomography coronary angiography (CTCA) has gained increasing attention in the clinical setting as a non-invasive substitute of coronary angiography, thanks to the remarkable improvement of its imaging quality (4). CTCA is now considered as an accurate diagnostic tool for the assessment of CAD severity. It has been demonstrated that the combination of CTCA-derived 3D arterial models with the application of computational fluid dynamics (CFD) can offer a non-invasive assessment of the hemodynamic status of the artery of interest with an acceptable accuracy when compared to the invasively measured FFR (5–13). The existing approaches follow the rationale that since flow and pressure are not known *a priori*, lumped parameter models of several factors such as the pressure of the systemic circulation and the resistance of the coronary microcirculation need to be coupled with the fluid domain of the epicardial arteries, resulting to laborious virtual FFR calculations that require a large computational time. Recently, the virtual functional assessment index (vFAI) has been introduced as one amongst several valid computational FFR surrogates [i.e. such as QFR (14), DEEPVESSEL-FFR (15), FFRangio (16) as well as the aforementioned functional assessment indices given above] in patients undergoing ICA (17) or CTCA (18, 19), being able to determine the functional severity of a coronary lesion in an arterial segment. The non-invasive FFR computation by CFD on CTCA images currently adopted in clinical practice (5) is an off-site assessment with a relatively long computational time and no substantial advantages compared to alternative on-site CTCA assessment approaches as recently reported (12).

In this study, we present a new approach for a really on-site and real-time, geometrically derived functional assessment of coronary stenosis, which can be performed both with CCTA or ICA datasets and both in case of stenosis involving a single coronary segment as well as a coronary bifurcation (excluding the common trunk and the common trunk bifurcation). One of the main advantages of the proposed method is its speed and the ability to perform it on-site. The overall diagnostic performance of the proposed method was tested in a CTCA patient dataset as well as in an ICA patient dataset and compared with traditional pressure-wire derived FFR measurements available in both datasets. Furthermore, we also examined the optimal simulation type for the SmartFFR calculation by comparing rigid wall simulations against fluid structure interaction simulations.

## Materials and Methods

### Study Population

**Figure 1:**
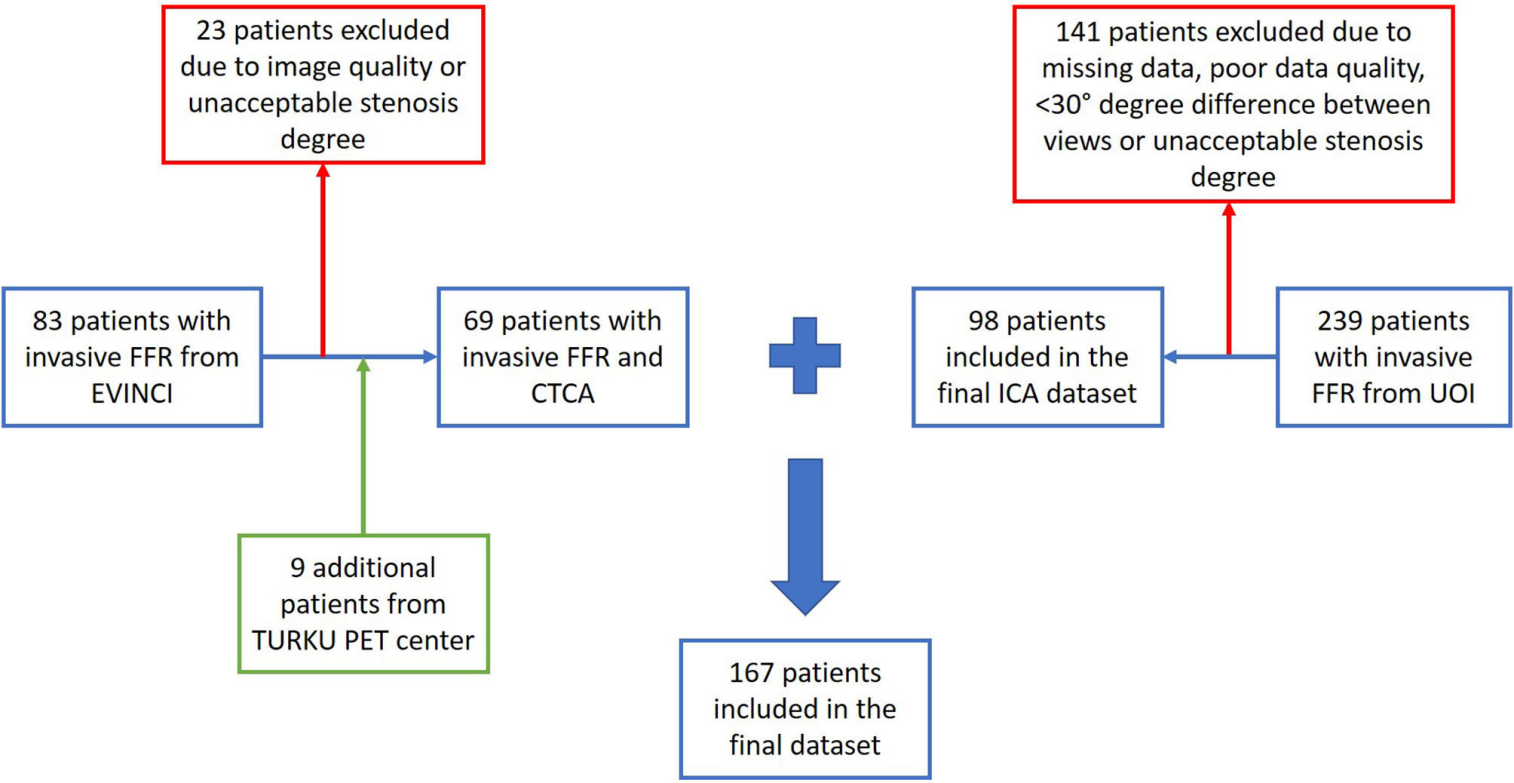
Enrollment and diagnostic procedures.

To obtain a suitable CTCA-derived dataset, data from the multicenter EVINCI (EValuation of INtegrated Cardiac Imaging for the Detection and Characterization of Ischemic Heart Disease) project, as well as from the SMARTool (Simulation Modeling of coronary ARTery disease: a tool for clinical decision support) project were used. The aforementioned projects complied with the Declaration of Helsinki. In the context of the EVINCI study (20), ethical approval was provided by each participating center and all subjects gave written informed consent. For the present study investigating anonymized imaging data, informed consent was waived. From the EVINCI and SMARTool populations, we chose 69 patients with intermediate (20–90%) pre-test probability of CAD (21) who underwent CTCA and ICA exams and had invasive FFR assessed in at least one major vessel. The exclusion criteria were previous acute coronary syndrome, left ventricular ejection fraction < 35%, cardiomyopathy, known CAD and more than moderate valve disease. An additional exclusion criterion was poor CTCA scan quality evaluated in a four levels scale (poor, satisfactory, good and excellent). Poor quality scans were excluded from our study. The final CTCA-derived dataset consisted of 88 major coronary arteries (Figure 1).

Regarding the ICA-derived dataset, a study population of 98 patients with stable or unstable angina or non-ST elevation myocardial infarction undergoing ICA and invasive FFR measurement at the University Hospital of Ioannina was retrospectively included in this study. The final ICA-derived dataset consisted of 114 major coronary arteries. Details on the coronary artery type distribution and patient demographics are presented in **Table 3**.

### CTCA Acquisition

CTCA was performed in all patients using ≥64-slice scanners. All the arteries which were used in the present study were reconstructed at mean diastole (70%-80% of R-R interval) (22). The slice increment was 0.6 mm, whereas the average slice thickness was 0.625 mm. The pixel spacing values varied due to the different scanners that were used throughout the multi-center EVINCI study. Nitrates were used to enhance the CTCA quality, whereas beta-blockers were used when necessary to reduce the heart rate in order to perform good quality examinations and nitrates were always used as described in the international guidelines (23).

### Invasive Coronary Angiography and FFR Acquisition

Standard techniques were used for the ICA acquisition with multiple projections. FFR was invasively measured after the intravenous administration of 140 μg/kg/min of adenosine, using a combo-wire (Volcano Corporation). Arterial segments stenoses with FFR values lower than 0.80 were defined as hemodynamically relevant. The ICA dataset was provided by the University hospital of Ioannina which included all the FFR measurements that were performed during a period of 4 years. The main reasons for the exclusion of ICA cases were the following: a) poor image resolution, b) < 30° angle difference between two ICA projections, c) presence of only one ICA projection, d) stenosis degree either < 30 or >90%.

### 3D Reconstruction

The reconstruction of the arterial models was performed with our in-house developed algorithms for the CTCA-derived dataset (24, 25) and for the ICA-derived dataset (26) and are both described in detail in the online Supplementary Material section. An expert interventional cardiologist (LL) performed the segmentation of the vessels of interest using the ICA images that contained the FFR wire within the vessel, in order to ensure the co-alignment of the FFR measured segment with the respective 3D reconstructed one.

### SmartFFR Calculation

**Figure 2:**
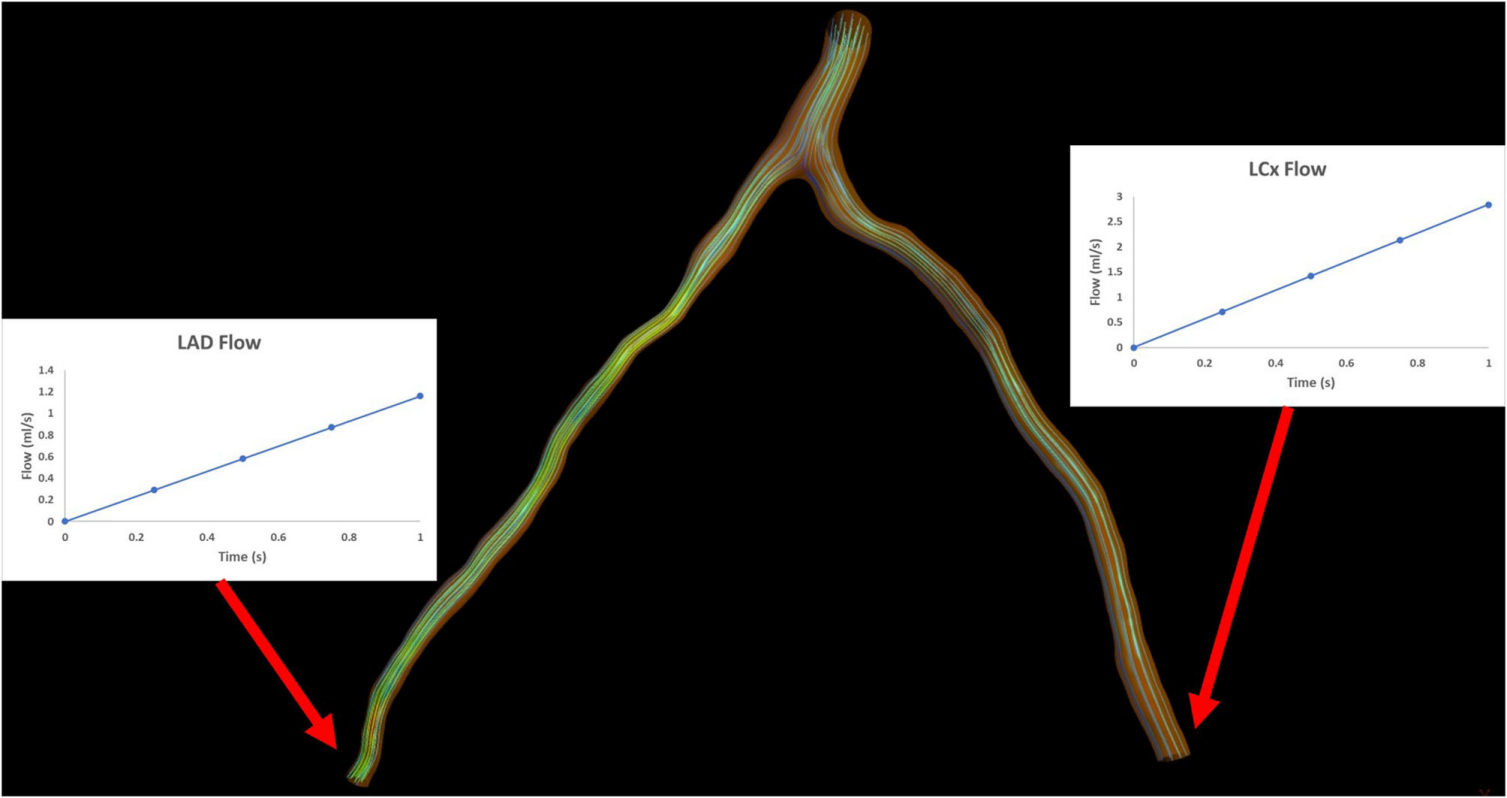
Illustration depicting the outlet boundary conditions for an arterial bifurcation.

In order to calculate SmartFFR, blood flow simulations are carried out on the reconstructed 3D models of the arteries of interest using the finite element method. The arterial lumen is discretized into tetrahedral finite elements of face size that ranges from 0.09–0.12 mm, as defined by a mesh sensitivity analysis, and the respective Navier-Stokes and continuity equations are then solved using ANSYS® CFX 16.2. The SmartFFR index is primarily based on the virtual functional assessment index (17) as an outcome, but it has some key points of deviation regarding the process with which the index is calculated, constituting the method more robust, faster and able to be applied on more than one segment at a time. A transient blood flow simulation is performed on the 3D reconstructed artery for which the boundary conditions which are applied in single-segment simulations are the following:

**Inlet**: an average static pressure of 100 mmHg is applied as inlet boundary condition.
**Outlet**: an increasing transient flow profile is applied as an outlet boundary condition. It consists of 4 timesteps of a duration of 0.25 s each and flow rate values are increasing from 1 to 4 ml/s with a step of 1 ml/s (Figure 2).
**Wall**: a no-slip and no-penetration boundary condition is applied at the arterial wall.

For each timestep, the P_d_/P_a_ value is calculated in order to construct the P_d_/P_a_ vs. flow curve. The calculated P_d_/P_a_ values for every timestep are then connected to create the appropriate patient-specific curve, using a smooth spline approximation with a total of 100 interpolation points using a dedicated script in MATLAB. The patient-specific curve is constructed for a flow range of 0–4 ml/s and the SmartFFR value is calculated by dividing the area under the patient-specific curve to the respective area under the curve of the respective healthy arterial segment (i.e. AUC = 4), following the vFAI rationale by Papafaklis et al. (17).

**Figure 3:**
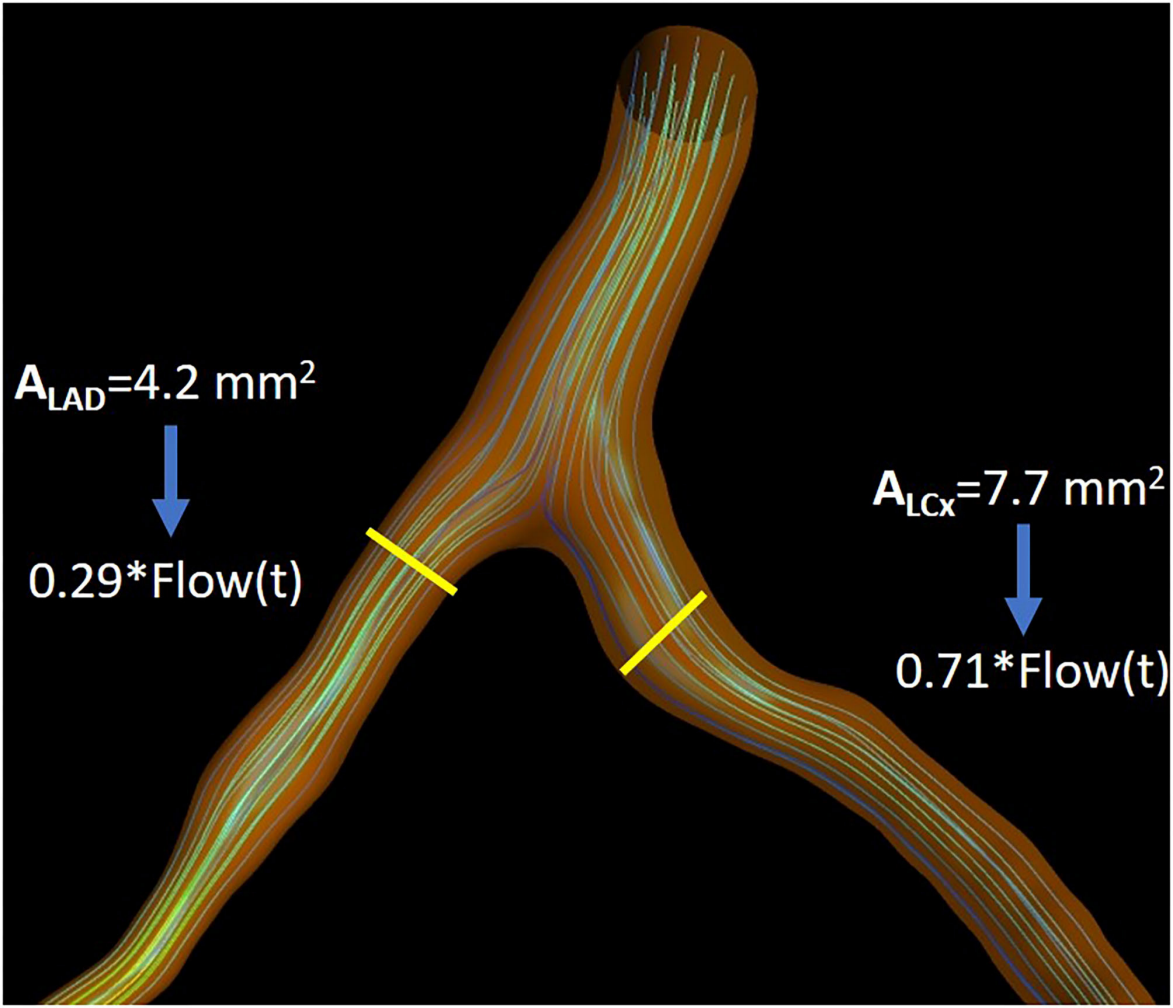
Illustration depicting the flow separation ratio as calculated for an indicative case, as calculated by Murray's law.

In the ICA-derived dataset, only the main vessel was reconstructed, whereas in the CTCA-derived dataset, when the simulation was performed in the left coronary artery system, the two main coronary arteries (LAD and LCx) were reconstructed. In order to calculate the SmartFFR in bifurcating arterial models, we first have to determine the flow ratio that enters each branch. The left descending and circumflex coronary arteries were evaluated by 3D reconstructed models and the flow ratio of each branch was determined at the level of the bifurcation involved. For the left vasculature, we assume a flow rate of 2 ml/s during rest that might be evenly distributed in the two main branches if we have an equal area at the inlet of the two branches (27). However, the patient-specific flow that enters each branch needs to be defined for every case. In order to do that, we apply Murray's law after having calculated the diameters and the areas of the two branches. Murray's law correlates the flow ratio that passes through the two branches with the respective diameters of the two branches. The aforementioned relation is given by:

(1) $$\begin{array}{l} \frac{q_{D2}}{q_{D1}}={\left(\frac{d_{D2}}{d_{D1}}\right)}^{3}, \end{array}$$

where *q*_*D*1_ and *q*_*D*2_ are the flows of branches 1 and 2 and *d*_*D*1_ and *d*_*D*2_, their respective diameters (Figure 3).

After having calculated the ratio, we then perform a transient blood flow simulation in the entire model with the following outlet boundary conditions:

**Outlets**: an increasing transient flow profile is applied as a boundary condition. However, in this case, we need to calculate the flow of each branch for each time step. The left main branch of the coronary vasculature has a total flow of around 2 ml/s during rest as it has been calculated through PET quantitative measurements. This is expected since both left main branches (i.e. Left Anterior Descending and Left Circumflex) average a mere 1 ml/s during rest (i.e. approximately equal to the respective flow during rest of the Right Coronary Artery). We assume that in a totally healthy left vasculature, we will have a peak hyperemic flow of 8 ml/s (i.e. 4 ml/s per branch) entering the left main stem [i.e. following the rationale of (28) stating that it is equal to the mean ± 2SD hyperemic flow increase in a normal artery] (29). Having this in mind, we create a transient flow of 4 timesteps of 0.25 s each with a total flow for each timestep from 0 to 8 ml/s. The outlet flow of each branch is calculated using the previously computed flow ratio and is applied for each timestep, respectively.

The inlet and wall boundary conditions were the same as in the single segment smartFFR calculation process. Flow is considered laminar, and blood is treated as a Newtonian fluid with density 1050 kg/m^3^ and dynamic viscosity 0.0035 Pa·s.

**Figure 4:**
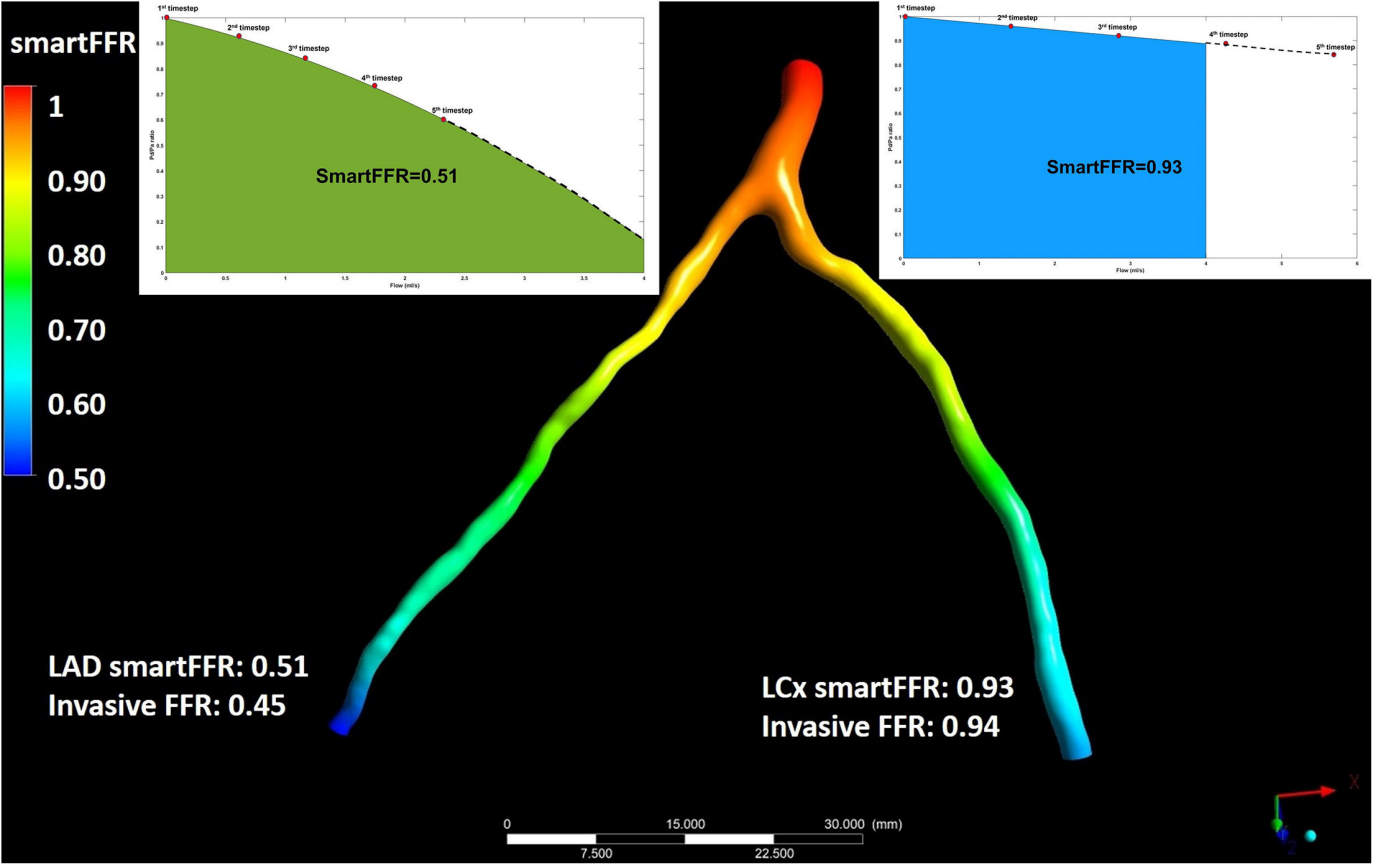
Illustration depicting the simultaneous SmartFFR calculation process for the two main branches of the left coronary vasculature (i.e. LAD and LCx). The dashed line after the last simulation timestep at the LAD branch was used to extrapolate the curve to reach the 4 ml/s mark, whereas for the LCx branch, the curve was interpolated to limit the curve to the 4 ml/s mark, respectively.

In order for the SmartFFR value to be calculated for each branch, we need to calculate the P_d_/P_a_ values for each time step at each branch. In order to do that, we first have to find the computed pressure at the inlet of each of the two branches, since this is the inlet pressure for each branch and not the overall inlet pressure at the left main stem. After having the P_d_/P_a_ values calculated for each timestep, we then build the respective P_d_/P_a_ vs. flow curves for each branch. In order to have a balanced universal value for each branch, we have to interpolate the curve of the branch with the higher flow up to a flow of 4 ml/s and extrapolate the respective curve of the second branch up that had the lower flow to the same value. The flow division is performed in order to apply physically valid boundary conditions on the two branches regarding the outlet, since the two branches cannot have an equal division of flow. SmartFFR is then calculated as the ratio of the area under the patient-specific curve divided by area under the curve of the respective healthy arterial segment for each branch (Figure 4). The SmartFFR values were calculated in a blind fashion from the actual FFR values.

### Effect of Fluid Structure Interaction (FSI) on SmartFFR

We have investigated the effect of different simulation methods on the calculated SmartFFR values. The whole process is described in detail in the online Supplementary Material.

### Statistical Analysis

The relationship between FFR and SmartFFR was quantified by calculating the Pearson's correlation coefficient. In order to assess the agreement between the two methods, the Bland–Altman plots and the corresponding 95% limits of agreement were used. A Receiver Operator Curve (ROC) analysis was performed to identify the cut-off values of the examined variables. The categorization of FFR and SmartFFR values was made using the cut-off value of 0.8 and the calculated cut-off from ROC curve for the FFR and SmartFFR (for each dataset separately), respectively. Sensitivity (SE), specificity (SP), positive predictive value (PPV), negative predictive value (NPV), and diagnostic accuracy (the percentage of patients correctly diagnosed by SmartFFR) were used to assess the performance of SmartFFR. *P* values < 0.05 were considered statistically significant. The comparison between the ROC curves was based on the DeLong method (MedCalc software).

## Results

From the CTCA-derived and ICA-derived datasets, 202 major coronary arteries (i.e. stenosis degree ranging between 30–90%), in which invasive FFR had been measured, were used to compute and validate the SmartFFR index. The ICA-derived dataset consisted of 114 major coronary vessels from 98 patients. Among the 114 arteries, 81 were LAD segments, 18 were LCx segments and the remaining 15 were RCA segments. 29 vessels (i.e. 25.4%) presented with a pathologic FFR value (i.e. FFR ≤ 0.80) and among these, sixteen arteries had FFR values within the so-called “gray zone” (i.e. FFR 0.75–0.80) (30).

In order to validate the efficacy of the bifurcation-based SmartFFR, we used the CTCA-derived dataset. The dataset consisted of 88 major coronary arteries. However, we must here state that the cases for which FFR measurements of both LAD and LCx branches (i.e. simultaneous SmartFFR calculation for two branches) were available were very few (i.e. nine cases). Twenty-seven cases (i.e. 30.7%) exhibited an ischemic FFR value (i.e. FFR ≤ 0.80) and from these, eleven cases were within the “gray zone”. In order to tackle this issue, we validated the method by comparing the SmartFFR value with the respective invasively measured FFR value of the branch that was available.

Strong correlation was observed between the two methods for the three (i.e. CCTA-derived dataset, ICA-derived dataset and overall dataset) datasets (R_CCTA_ = 0.86, p_CCTA_ < 0.0001, R_ICA_ = 0.84, p_ICA_ < 0.0001 and R_overall_ = 0.833, p_overall_ < 0.0001, respectively) and good agreement was observed by the Bland-Altman method of analysis (**Figure 6**). For the ICA-derived dataset there was a slight overestimation of FFR by SmartFFR in this case with a mean difference of 0.024 ± 0.051 (*p* < 0.0001). The corresponding limits of agreement were −0.012 to 0.08 with 95% confidence intervals −0.14 to −0.11 for the lower limit and 0.06 to 0.09 for the upper limit, respectively. For the CCTA-derived dataset there was a slight underestimation of FFR by SmartFFR with a mean difference of 0.006 ± 0.053 (*p* = 0.26). The corresponding limits of agreement were from−0.098 to 0.11 with 95% confidence intervals −0.1150 to −0.08135 for the lower limit and −0.1150 to −0.08135 for the upper limit, respectively. Finally, for the overall dataset, there was a slight overestimation of FFR by SmartFFR in this case with a mean difference of 0.007 ± 0.053 (*p* < 0.0001). The corresponding limits of agreement were −0.0147 to 0.00016 with 95% confidence intervals −0.1251 to−0.09966 for the lower limit and 0.085 to 0.11 for the upper limit, respectively.

The interobserver agreement for SmartFFR measurements was tested in 20 randomly selected coronary vessels reconstructed from ICA (12 LAD, 4 LCx and 4 RCA, respectively) and 20 vessels reconstructed by CTCA (13 LAD, 3 LCx and 4 RCA, respectively). Strong agreement was found between the two observers for the CTCA dataset (mean difference = −0.007 ± 0.01, *p* = 0.0068), as well as for the ICA dataset (mean difference = −0.009 ± 0.018, *p* = 0.04). The intraobserver agreement for SmartFFR measurements was tested in the same randomly selected set of 40 vessels (20 vessels reconstructed from ICA and 20 vessels reconstructed from CTCA, respectively). Excellent agreement was observed in the intraobserver variability analysis for the CTCA-derived dataset (mean difference = −0.002 ± 0.006, *p* = 0.16), as well as for the ICA-derived dataset, respectively (mean difference = −0.0035 ± 0.006, *p* = 0.15).

### Diagnostic Accuracy of SmartFFR (Overall Dataset)

**Figure 5:**
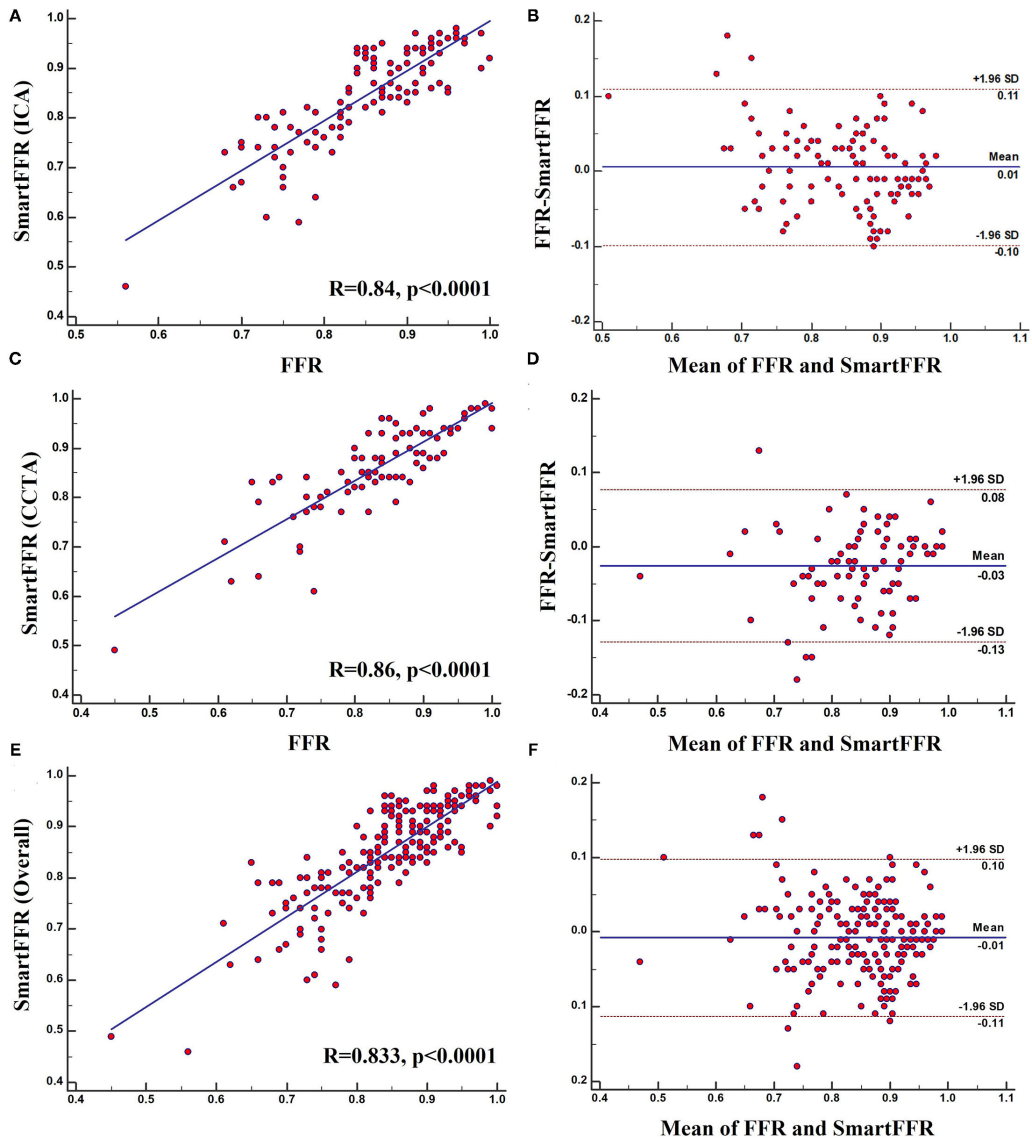
**(A)** Regression plot and **(B)** Bland-Altman plot comparing SmartFFR to the invasively measured FFR (ICA Dataset). **(C)** Regression plot and **(D)** Bland-Altman plot comparing SmartFFR to the invasively measured FFR (CTCA Dataset). **(E)** Regression plot and **(F)** Bland-Altman plot comparing SmartFFR to the invasively measured FFR (Overall dataset).

The optimal SmartFFR cutoff value for identifying a functionally significant stenotic segment with an FFR value ≤ 0.80 was ≤ 0.83 from receiver operator curve (ROC) analysis (Figure 5). The overall diagnostic performance of SmartFFR using the calculated optimal threshold but also the established FFR threshold of 0.80 is presented in Table 1 (AUC = 0.956, *p* < 0.001).

**Table 1:** Diagnostic performance of SmartFFR for the overall dataset, the ICA-derived dataset and the CCTA-derived dataset, for the optimal thresholds as calculated by the Youden index and for the established FFR threshold of 0.80.

	**FFR ≤ 0.80**								
	**Accuracy (%)**	**Sensitivity (%)**	**Specificity (%)**	**PPV (%)**	**NPV (%)**	**TP**	**TN**	**FP**	**FN**
SmartFFR ≤ 0.83 Overall dataset	88.1	94.6	85.6	71.6	97.7	53	125	21	3
SmartFFR ≤ 0.80 (Overall dataset)	89.1	76.8	93.8	82.7	91.3	43	137	9	13
SmartFFR ≤ 0.81 (ICA dataset)	91.2	96.6	89.4	75.7	98.7	28	76	9	1
SmartFFR ≤ 0.80 (ICA dataset)	91.2	89.7	91.8	78.8	96.3	26	78	7	3
SmartFFR ≤ 0.83 (CTCA Dataset)	90.9	88.9	91.8	82.8	94.9	24	56	5	3
SmartFFR ≤ 0.80 (CTCA Dataset)	86.4	63	96.7	89.5	85.5	17	59	2	10

### Diagnostic Accuracy of SmartFFR (ICA Dataset)

In the ICA-derived dataset, the optimal SmartFFR cutoff value for identifying a functionally significant stenotic segment with an FFR value ≤ 0.80 was ≤ 0.81, deriving from the receiver operator curve (ROC) analysis (Figure 5). The overall diagnostic performance of SmartFFR using the calculated optimal threshold but also the established FFR threshold of 0.80 is presented in Table 1 (AUC = 0.975, *p* < 0.001).

### Diagnostic Accuracy of SmartFFR (CTCA Dataset)

In the CTCA-derived SmartFFR analysis, the optimal SmartFFR threshold to identify a functionally significant stenotic segment with FFR ≤ 0.80 was SmartFFR ≤ 0.83, as dictated by the receiver operator curve (ROC) analysis (Figure 5). The overall diagnostic performance of SmartFFR is presented in Table 1 (AUC = 0.952, *p* < 0.001).

### SmartFFR and Type of Simulation

**Figure 6:**
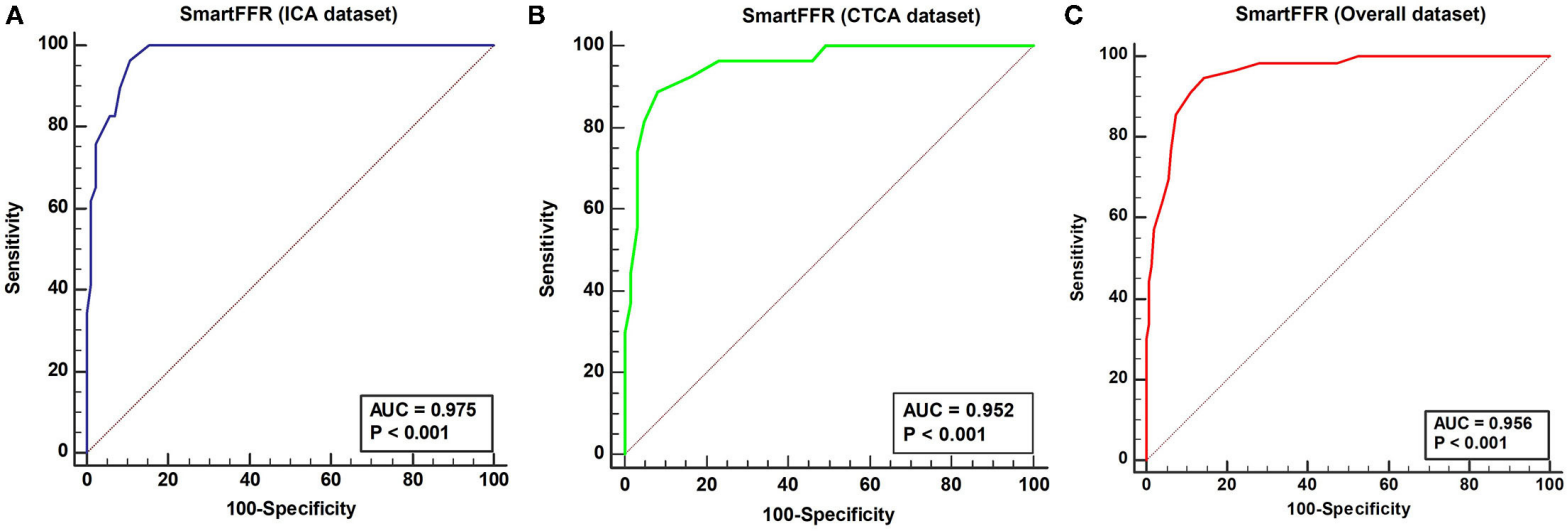
ROC curve depicting the diagnostic performance of SmartFFR for **(A)** ICA dataset (95% CI 0.91–0.98), **(B)** CTCA dataset (95% CI 0.88–0.99) and **(C)** Overall dataset (95% CI: 0.92 to 0.98).

To assess the possible effect of the simulation type on SmartFFR, a rigid wall or a FSI simulation model were used to compute SmartFFR in 25 coronary segments. The average SmartFFR for the rigid wall simulations for the 25 segments was 0.838 ± 0.19 whereas, for the FSI simulations the average SmartFFR was 0.848 ± 0.19, respectively. Strong correlation was found between the two simulation methods presenting with almost identical SmartFFR values (r = 0.99, *p* < 0.0001) (Figure 6).

**Figure 7:**
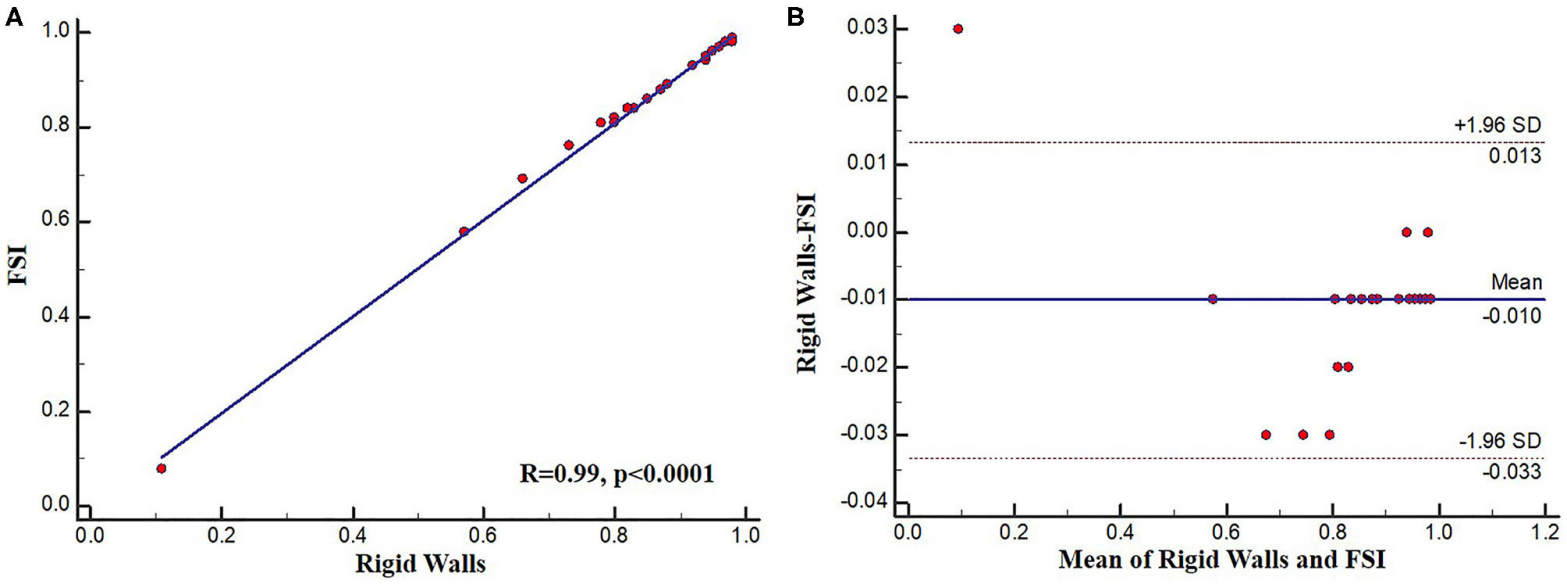
**(A)** Regression plot and **(B)** Bland-Altman plot comparing FSI and rigid wall SmartFFR calculations.

Excellent agreement was also found for the two methods of simulation with a mean difference of −0.010000 ± 0.012 as calculated by the Bland-Altman method of analysis (Figure 7). The upper limit was 0.0133 with 95% CI from 0.0048 to 0.022, whereas the lower limit was −0.033 with 95% CI from −0.042 to −0.025.

## Discussion

**Figure 8:**
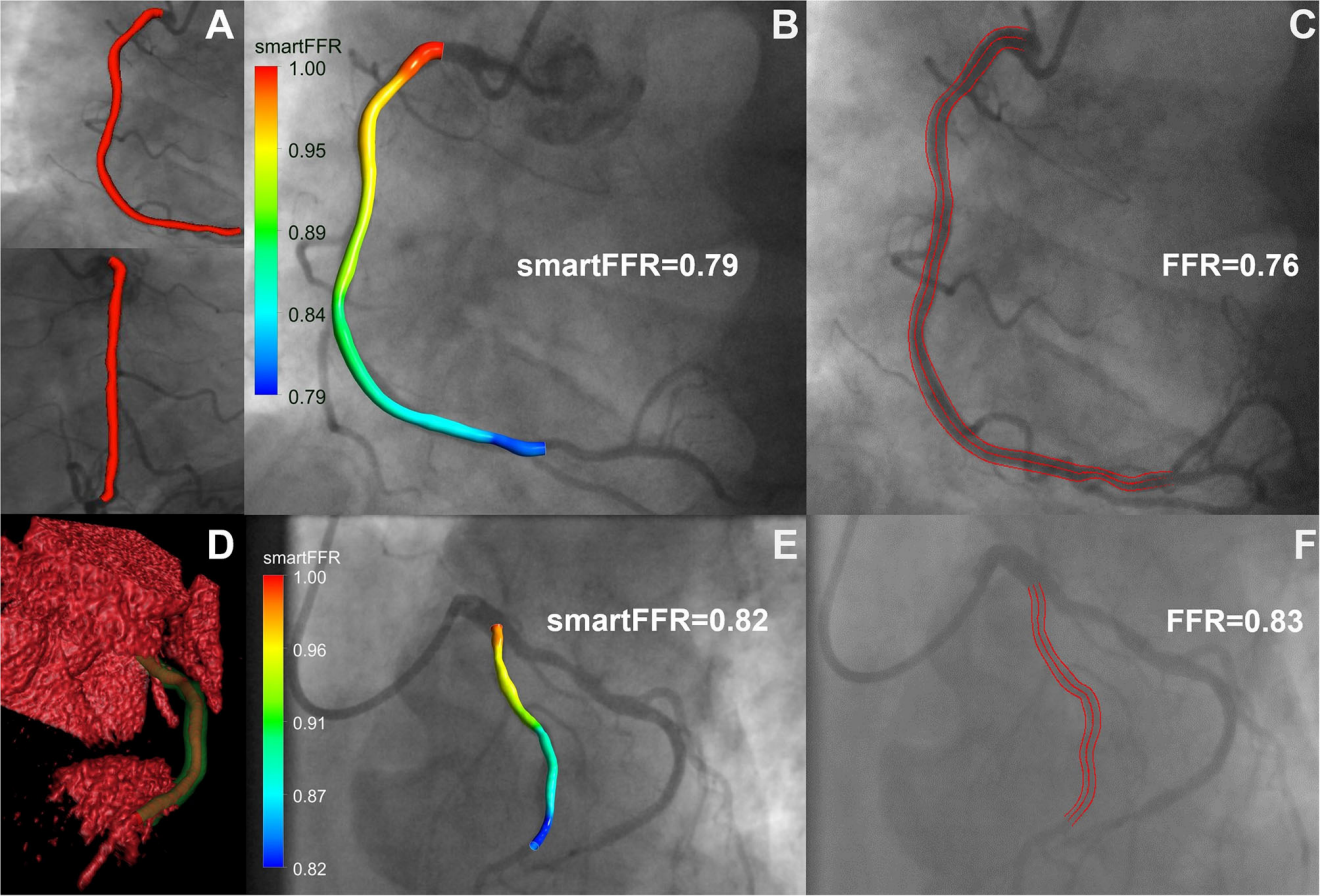
Top row: ICA-derived model, Bottom row: CCTA-derived model. **(A)** 3D reconstructed ICA-derived model back-projected on the two utilized angiographic views using our in-house developed software platform. **(B)** ICA-derived 3D model with smartFFR value, back-projected on the respective angiographic image, **(C)** ICA image with respective invasively measured FFR value and delineation of the segment of interest, **(D)** Volume rendered 3D reconstructed model with our in-house developed software platform. The outer wall is depicted in transparent green and the lumen in orange. **(E)** CCTA-derived 3D model with smartFFR value, back-projected on the respective angiographic image **(F)** ICA image of the CCTA model with respective invasively measured FFR value and delineation of the segment of interest.

In this study, we have demonstrated the efficacy of our newly proposed SmartFFR index in assessing the hemodynamic significance of coronary stenoses within a matter of minutes, using either the most well-known non-invasive cardiac imaging modality (i.e. CTCA), or the most-commonly used invasive coronary imaging modality (i.e. ICA) (Figure 8). We observed that SmartFFR values from the ICA-derived dataset had a slightly inferior correlation to the invasively measured FFR than those from the CTCA-derived dataset, but had slightly increased accuracy and sensitivity, possibly due to the higher spatial resolution of ICA. More specifically, in the overall dataset, SmartFFR matched the values of the invasively measured FFR closely, having sensitivity and specificity of 94.6 and 85.6%, respectively, using the computed cutoff value of ≤ 0.83 to identify stenoses of FFR ≤ 0.80 (Table 1).

Several studies have already demonstrated the efficacy of CTCA-derived or ICA-derived functional indices to identify ischemic lesions with the aid of CFD simulations (8, 31). In the first studies investigating the possible application of CTCA-derived computational FFR measurements, the agreement between FFR_CT_ and the invasively measured FFR was rather modest (32). However, by gaining the ability to create far more complex models of the coronary vasculature that included vascular microcirculation, the accuracy of FFR_CT_ was significantly improved over the past years and many studies demonstrated the efficacy of the method. The results of the present study indicate the efficacy of a new method, SmartFFR, to identify hemodynamically significant stenoses. Compared to the previously validated virtual Functional Assessment Index (17) which is the foundation for SmartFFR, SmartFFR required a lower total computational time, since only one blood flow simulation is needed (Table 2). Furthermore, SmartFFR allows for the simultaneous functional assessment of at least two vessels and could even allow for the assessment of more than two branches. When compared to other virtual indices, SmartFFR exhibits similar or even superior diagnostic performance having a diagnostic accuracy, sensitivity, specificity, PPV and NPV of 88.1, 94.6, 85.4, 71.6, and 97.7%, regarding the overall dataset (Table 1). Furthermore, SmartFFR can be calculated on a simple personal computer on-site, without the need of a dedicated core-laboratory and the total process time, along with the required 3D reconstruction time does not exceed an average of 10 minutes, depending on the available imaging modality (Table 2).

**Table 2:** Average required time for SmartFFR and vFAI.

**Imaging modality**	**Reconstruction time**	**Mesh generation**	**SmartFFR (per bifurcation)**	**vFAI (per segment)**
ICA	~3 min	~3 min	~3 min	~7 min
CTCA	~1-2 min	~3 min	~3 min	~7 min

### Study Limitations

Our study included a retrospective analysis of two imaging datasets including either invasive or non-invasive coronary angiographies. The rather limited number of patients included in the CTCA-derived dataset is a limitation. We tested the efficacy of the multi-vessel SmartFFR only on the left coronary system of the CTCA patients that had invasive FFR measurements available for the LAD, the LCx or both, since this was the only way to validate the efficacy of the method. Unfortunately, there was a lack of simultaneous invasive FFR measurements in two branches (only 9 cases had simultaneous invasive FFR measurements for the LAD and the LCx branch, respectively), which constitutes a limitation of our study. Even in this rather modest sample though, SmartFFR matched the invasively measured FFR values rather well, discriminating the hemodynamically significant stenoses with good accuracy. We should also mention that SmartFFR was tested also in a single-vessel manner in the CTCA dataset for the RCA cases that were examined. However, the majority of the lesions were located at the left coronary vasculature (i.e. ~80%) which is also a limitation of the examined dataset. Furthermore, the lack of diastolic blood pressure data did not allow us to substitute the universal inlet pressure value with a patient-specific blood pressure value, a methodological step that will further enhance the SmartFFR methodology. Moreover, Murray's law is generally used for idealized laminar flows and in cases of severe stenoses it might not be most suitable. However, we used laminar flow assumptions throughout the entire dataset in order to preserve consistency through the simulations. Finally, we must also mention the rather limited number of marginal cases (i.e. within the so-called FFR “gray-zone”), cases that are usually most challenging in terms of accuracy, even for the invasive FFR measurement (Table 3). We are currently working on broadening our validation dataset for the multi-vessel SmartFFR analysis, a non-trivial task though, since multiple invasive FFR measurements are required in the left coronary vasculature.

**Table 3:** Patient demographics and vessel characteristics.

**Cardiovascular risk factors**	
Age	63.1 (± 7.6)
Female	59 (29%)
Male	143 (71%)
BMI, kg/m^2^	27.8 (± 4.7)
Body Mass, kg	82.1 (± 16.9)
Diabetes (*N*, %)	47 (23.2%)
Smoker during past year (*N*, %)	41 (20.3%)
Hypertension (*N*, %)	138 (68.3%)
Hypercholesterolemia (*N*, %)	138 (68.3%)
**Coronary vessels (***N***, %)**	
Right coronary artery	39 (19.3%)
Left anterior descending	131 (64.9%)
Left Circumflex	32 (15.8%)
**Severity of coronary lesions at ICA (***N***, %)**	
Stenosis 30–49%	89 (44.3%)
Stenosis 50–70%	69 (34.1%)
Stenosis 70–90%	44 (21.6%)
**FFR categories (***N***, %)**	
FFR ≤ 0.75	35 (17.3%)
FFR > 0.75 and ≤ 0.8	21 (10.4%)
FFR > 0.8	146 (72.3%)

## Conclusions

We have demonstrated the efficacy of SmartFFR to discriminate hemodynamically significant stenoses in either CTCA-derived or ICA-derived coronary 3-dimensional models on-site with relatively fast computational time and low computational cost. SmartFFR correlated well with the invasively measured FFR, which is the gold standard in the functional assessment of coronary stenoses.

## Data Availability Statement

The raw data supporting the conclusions of this article will be made available by the authors, without undue reservation. Requests to access data should be directed to Silvia Rocchiccioli, silvia.rocchiccioli@ifc.cnr.it, Lampros K. Michalis, lamprosmihalis@gmail.com.

## Ethics Statement

The studies involving human participants were reviewed and approved by each participating center (National Research Council, University of Turku, University of Zurich, Fondazione Toscana Gabriele Monasterio, Warsaw National Institute of Cardiology) through the approval of the clinical study by the Ethics Committee Vast Area Northwest of Tuscany (CEAVNO), Pisa, Italy, and all subjects gave written informed consent. Our clinical study follows the declaration of Helsinki. The patients/participants provided their written informed consent to participate in this study.

## Author Contributions

PS performed the computational work, the concept of the method, and composed the manuscript. LL performed the annotation of the ICA dataset. AS contributed in the CCTA reconstruction and manuscript editing. GR, SK, and KS created the CCTA and the ICA reconstruction algorithms. CA and AC contributed in editing the manuscript. SR, GP, and OP contributed in the CCTA dataset. DN contributed in the CCTA dataset and the manuscript editing. KN and LM contributed in the ICA dataset. MP contributed with the initial vFAI methodology. DF contributed in the overall concept of the manuscript and the final editing of the manuscript. All authors contributed to the article and approved the submitted version.

## Conflict of Interest

The authors declare that the research was conducted in the absence of any commercial or financial relationships that could be construed as a potential conflict of interest.

## Publisher's Note

All claims expressed in this article are solely those of the authors and do not necessarily represent those of their affiliated organizations, or those of the publisher, the editors and the reviewers. Any product that may be evaluated in this article, or claim that may be made by its manufacturer, is not guaranteed or endorsed by the publisher.
