# Supplementary material for: SmartFFR, a New Functional Index of Coronary Stenosis: Comparison With Invasive FFR Data
Source: Front Cardiovasc Med. 2021 Aug 17;8:714471. doi: 10.3389/fcvm.2021.714471 (PMC8418116; doi:10.3389/fcvm.2021.714471)
Supplement: Supplementary file 1 [file Data_Sheet_1.docx]

**CTCA 3D Reconstruction**

Our in-house developed algorithm consists of seven steps. The Right Coronary Artery (RCA) was reconstructed as a segment since the side branches of the RCA are not usually included in an FFR assessment due to their small size, compared to the main RCA branch. Regarding the left vasculature, we reconstructed the Left Main stem along with the two main branches that follow it (i.e. Left Anterior Descending and Left Circumflex). In the first step, the acquired CTCA images are initially pre-processed to detect possible vessel silhouettes. In the second step, a rough initial centerline extraction approach of the vessel is applied. In the third step, a weight function for the arterial lumen, the outer wall and the plaques is estimated. In the fourth step, an extension of active contour models for lumen segmentation is applied. In the fifth and sixth steps, as in the previous step, a level set methodology for the outer wall and the plaque segmentation, respectively, is applied. Finally, in the last step, the 3D surfaces for the lumen, the outer wall and the plaques are constructed.

**ICA 3D Reconstruction**

The 3D reconstruction of the arteries of interest using ICA data was performed using our in-house developed software. Briefly, the user manually segments the luminal borders in each of the two angiographic projections. Then the centerline of the vessel of interest is automatically extracted in each of the two projections and *n* equidistant points in each centerline are created. For each centerline the perpendicular line in each of the *n* points is computed. In each projection the perpendicular lines intersect the silhouettes of the vessel projections in two points, having a distance $r_{1}^{P1}$ and $r_{1}^{P2}$ from the first and second silhouette, respectively. Then for each of the *n* points, *n* circles (contours) are computed with a radius that is calculated as:

$r_{1}=\frac{r_{1}^{P1}+r_{1}^{P2}}{2}$.  (1)

Finally, the 3D path is reconstructed and the created contours are placed perpendicularly on the 3D centerline, generating the 3D arterial model.

**Effect of Fluid Structure Interaction (FSI) on SmartFFR**

In order to achieve that, we used 25 arterial segments on which we had previously calculated SmartFFR, and we performed FSI blood flow simulations. In these simulations, the arterial wall was modelled as a homogenous elastic material, the material properties of which are given in Table 1. Regarding the boundary conditions of the arterial wall, the distal ends of the artery acted as fixed supports in order to constrain excessive movement of the arterial model. The inner boundary of the arterial wall acted as the FSI interface between the wall and the arterial lumen. In order to achieve convergence for the FSI problems, we used an inflation meshing method for both the wall and the arterial lumen at their common interface, which consisted of 5 layers of brick elements for each domain, respectively.

Table 1: Material properties of the arterial wall

| **Elastic Modulus** | **Poisson’s Ratio** | **Density** |
| --- | --- | --- |
| 1.06 MPa | 0.45 | 1120 kg/m^3^ |
